# An Initiative to Prevent Newborn Drops through the Implementation of a Prevention Bundle at a Children’s Hospital

**DOI:** 10.1097/pq9.0000000000000836

**Published:** 2026-07-29

**Authors:** Holly M. Birkinshaw, Ashley R. Owens, Aries C. Gilchrist, Jennifer Barrows, Jennifer Lusk

**Affiliations:** From the * Medical Unit, Children’s Hospital of Orange County (CHOC), part of Rady Children’s Health, Orange, Calif.; † CHOC, part of Rady Children's Health, Orange, Calif.; ‡ Department of Pediatrics, UC Irvine, Irvine, Calif.

## Abstract

**Introduction:**

Newborn drops are a problem in hospitals across the United States. This type of fall, characterized by an infant slipping from the arms or lap of a parent or caregiver, can have severe consequences and result in emotional distress for families. The lack of a comprehensive approach to preventing newborn drops at our children’s hospital highlighted an urgent need for action. Although existing research has focused on maternal-child acute care units, a notable gap remains in evidence-based solutions for children’s hospitals. We developed a baby drop prevention bundle to address this problem.

**Methods:**

We used improvement science to implement and evaluate the bundle across the hospital. A Plan–Do–Study–Act model guided iterative cycles of improvement. The bundle included staff and caregiver education, frequent intentional safety rounding for newborns aged 30 days or younger, an infant fall risk assessment, a supportive pillow for newborn holding and feeding, protected maternal rest periods, and standardized reporting and debriefing of infant drops and near misses. The primary outcome measure was the incidence of newborn drops; the process measure was compliance with the bundle, and an additional measure was near-miss events.

**Results:**

Since implementing the baby drop prevention bundle, there have been 0 incidents of newborn drops. We have observed 10 near-miss events since May 2025. Compliance reached 100% with the rounding plan and 90% with the use of the supportive pillow during 10 months.

**Conclusions:**

A baby drop prevention bundle likely reduced the risk of newborn drops and promoted a culture of safe sleep at a children’s hospital.

## INTRODUCTION

Falls among newborns in the hospital are a significant concern, with an estimated 600–1,600 falls reported annually in the United States.^1^ In this article, the term fall will refer to infant drops to align with what has been reported in the literature. Infant drops are defined as a sudden, unintentional descent in which the infant falls to the floor or another surface, potentially causing injury.^2^ These incidents occur when an infant slips from a caregiver’s arms or lap.^2^ The consequences of such falls can be severe, leading to serious injuries such as skull fractures and intracranial hemorrhages.^3–7^ Moreover, the emotional distress resulting from these falls can affect newborns’ families as well as their healthcare team.^3^

Various maternal risk factors contribute to newborn falls, including fatigue,^1,4,5,7–12^ breastfeeding,^12–16^ extended postpartum recovery,^13,14^ pain medication use,^5,11–13,16,17^ and prior near-miss events.^16^ The time of day also plays a role, as most falls occur late at night or early in the morning.^1,4,5,7,9,11,13,14,16^ Given that many falls happen when caregivers fall asleep while holding or feeding their babies,^3,8^ it is crucial to address caregiver fatigue. Potential approaches include promoting caregiver rest,^4,7,13,18–21^ using supportive devices or pillows during feeding and holding,^11,22^ and ensuring an alert adult remains in the room.^11,17,18,20,23^

A multifaceted intervention is necessary to address the complexities surrounding newborn falls.^18^ Recommended strategies include educating parents, caregivers, and staff about fall risks, enhancing staff rounds, using risk assessment tools, creating formal pledges or contracts, displaying safety reminders, auditing safe sleep practices, monitoring near-miss events, standardizing incident reporting, and conducting debriefings after fall events and near misses. These evidence-based intervention strategies are summarized in Table 1.

**Table 1.** In-hospital Infant Fall Prevention Strategies

	References																		
	Helsley et al^1^	Hughes Driscoll et al^4^	Wallace et al.^7^	Bittle et al^8^	Carr et al.^9^	Karlsson et al^10^	Miner^11^	Whatley et al^12^	Galuska^13^	Unal et al^16^	Ainsworth et al^17^	Association of Women’s Health, Obstetric and Neonatal Nurses ^18^	The Joint Com-mis-sion^19^	Knipper et al^20^	Krening et al^22^	Lipke et al^23^	Matteson et al^24^	Pahuja^25^	Slogar et al^21^
Promoting caregiver rest		**·**	**·**						**·**			**·**	**·**	**·**					
Using a supportive pillow for newborn feeding or holding							**·**								**·**				
Having an alert adult remain in the room								**·**			**·**	**·**		**·**		**·**			
Educating parents, caregivers, and staff about infant fall risk	**·**	**·**	**·**	**·**	**·**		**·**		**·**		**·**	**·**	**·**	**·**		**·**	**·**	**·**	**·**
Increasing frequency of rounds or conducting safety rounds	**·**	**·**	**·**	**·**	**·**		**·**		**·**		**·**	**·**	**·**	**·**		**·**		**·**	**·**
Using a fall risk assessment tool			**·**	**·**	**·**			**·**				**·**	**·**	**·**	**·**		**·**	**·**	**·**
Creating a formal pledge or contract for infant safety	**·**	**·**	**·**		**·**	**·**	**·**		**·**							**·**			
Displaying safety reminders in the room, unit, and/or crib		**·**	**·**		**·**		**·**	**·**	**·**		**·**		**·**	**·**		**·**		**·**	
Auditing safe sleep practices							**·**					**·**							
Monitoring near-miss events								**·**		**·**				**·**					**.**
Standardized reporting of infant falls	**·**		**·**		**·**		**·**	**·**			**·**	**·**	**·**			**·**		**·**	**·**
Debriefing staff on infant falls and near misses	**·**		**·**		**·**		**·**				**·**	**·**	**·**	**·**		**·**	**·**	**·**	**·**

Existing research has primarily focused on maternal newborn units, revealing a gap in evidence specific to pediatric hospitals, where newborns face unique vulnerabilities due to maternal fatigue, postpartum recovery, and medical interventions.^8,9,20^ In response to 2 incidents at our organization in 2019, we focused on promoting safe sleep to reduce the risk of newborn falls. Although we established a safe sleep culture, with no falls reported in 2021, 2 incidents occurred in 2022, highlighting the need for additional interventions. Thus, this project aimed to decrease infant falls to 0 by the end of fiscal year 2023 through implementation of a comprehensive baby drop prevention bundle for newborns aged 30 days or younger admitted to our children’s hospital.

## METHODS

**Fig. 1.**
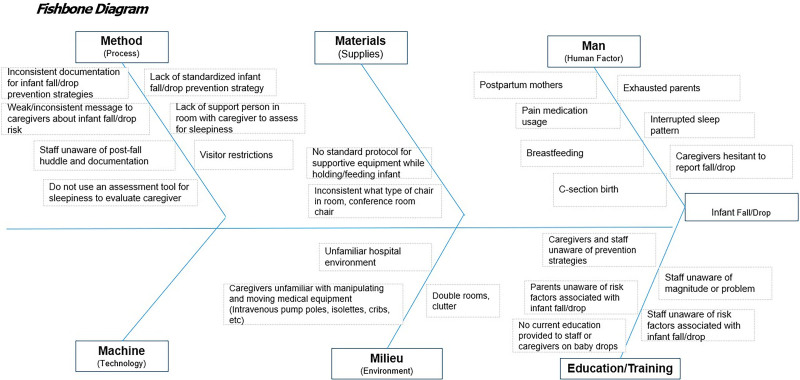
Fishbone diagram of factors contributing to infant falls/drops.

**Fig. 2.**
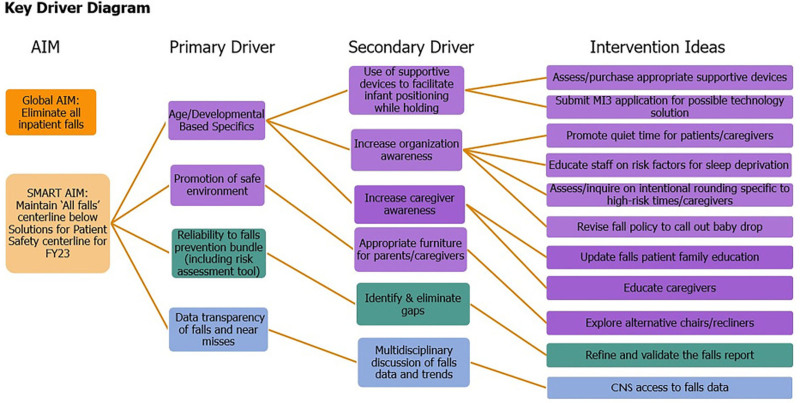
Key driver diagram of aim, drivers, and interventions to prevent falls.

This quality improvement (QI) project was implemented at an urban freestanding children’s hospital in the western United States, on a 48-bed acute care medical unit. We used the Institute for Healthcare Improvement model for QI methodology, which emphasizes thorough problem definition and understanding of the current state to inform effectively targeted and contextually appropriate interventions.^26^ A workgroup of key stakeholders was established in the Fall of 2022, including members from the quality department, leadership, clinical nurse specialists (CNSs), and clinical educators from the neonatal intensive care unit and medical unit. The workgroup reviewed best practices and conducted a gap analysis, using staff surveys and bedside audits to assess the baseline knowledge and practices related to safe sleep. A fishbone diagram was developed to determine the root causes that can lead to a newborn’s fall (Fig. 1). This analysis was used to create a key driver diagram, which helped establish workflows (Fig. 2). This QI project was deemed exempt from institutional review board approval at our organization.

### Interventions

**Fig. 3.**
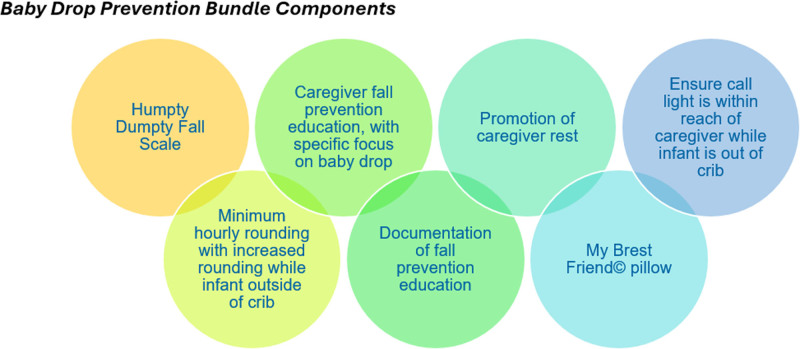
Baby drop prevention bundle components. Elements of the baby drop prevention bundle include the following: (1) HDFS: a validated tool to assess patient fall risk. (2) Caregiver education upon admission, transfer of care, and as needed, including a fall prevention handout. (3) Protected quiet hours and continually partnered with caregivers to promote rest. (4) Ensure the call light is within reach while the infant is out of the crib to promote caregiver communication with staff. (5) The minimum hourly rounding interval should be increased to every 30 minutes to enhance situational awareness and facilitate follow-up during high-risk periods when infants are out of the crib. (6) Documentation of fall prevention education during admission, transfer of care, and as needed for reinforcement. (7) Adopting the use of the My Brest Friend pillow to provide additional support for caregivers’ arms and ensure optimal infant positioning.

We developed a baby drop prevention bundle for newborns aged 30 days or younger (Fig. 3). This specific age range was chosen to align with the findings from previous fall events, where all patients were noted to be younger than 30 days. The bundle incorporated existing organizational practices, such as conducting risk assessments using the Humpty Dumpty Fall Scale (HDFS)^27^ and ensuring call lights were within caregivers’ reach. The HDFS is an evidence-based tool that comprises 7 items: age, sex, diagnosis, cognitive impairments, environmental factors, medication usage, and response to surgery, sedation, or anesthesia. New bundle elements focused on family and staff education, intentional safety rounding, increased attention to maternal and environmental factors, and the use of supportive pillows during feeding and holding. These components, detailed in the following sections, were added to the organizational fall policy in April 2023.

#### Parent and Caregiver Education

Parents and caregivers received infant safety education upon admission, during transfers of care, and as needed to reinforce learning. Education focused on using crib side rails, increased staff rounding, call light usage, and speaking up when the caregiver was tired. The fall safety handout was redesigned in collaboration with the Patient and Family Advisory Council to incorporate infant drop prevention. (**See figure 1, Supplemental Digital Content 1**, which displays a patient and family education handout on preventing infant falls/drops, https://links.lww.com/PQ9/A699.) This council comprises parents of former patients, patient and family experience representatives, and a social worker. Previously, the handout included typical elements of fall prevention for older children, such as nonskid footwear. The revisions added specific guidance to address infant fall risk factors and prevention strategies. Staff documented caregiver education in the electronic health record.

#### Staff Education

The medical unit nursing staff received education on risk factors associated with infant falls and preventive strategies during staff meetings in February 2023. The CNS and clinical nurse educator presented an instructional slide deck with opportunities to ask questions and provide feedback. Through an online learning module, we expanded education in March 2023 to unit assistants, respiratory care practitioners, patient transporters, discharge nurse navigators, and emergency department technicians. The clinical nurse educator and CNS participated in staff rounding to reinforce learning, clarify bundle elements, and optimize compliance. Additional education was provided to nursing assistants in August 2023 to offer a supportive pillow as part of the admission process for newborns aged 30 days or younger. Infant fall risk and prevention strategies were integrated into the onboarding program for new staff and the organization’s annual compliance modules to ensure all staff members were adequately informed.

#### Intentional Rounding

Intentional rounding focused on assessing environmental safety and caregiver alertness. The family and staff created an operational rounding plan for each shift by partnering to notify staff when infants were out of their cribs. Staff increased the frequency of rounding to every 30 minutes during this time. This time interval was selected because it coincided with the duration of a typical infant feeding period. At times, staff raised concerns about the feasibility of completing the rounding. To address these concerns, staff received additional training on the rationale for frequent rounding, and charge nurses considered the increased staffing demands when assigning patient care.

#### Supportive Pillow

A supportive pillow (My Brest Friend; Zenoff Products, San Marcos, CA), specifically designed for holding and feeding infants, was adopted from the neonatal intensive care unit by the medical unit. During rounds, it became evident that caregivers often underused or declined to use the pillow. To address this barrier, we included the supportive pillow as a standard part of the admission process for eligible newborns. In August 2023, the patient and family advisory council helped identify solutions to address the underusage of the supportive pillow. This intervention resulted in the development of updated educational materials that included visual aids and the rationale for their use.

#### Maternal and Environmental Factors

Promoting caregiver rest was essential. Staff advocated for protected rest periods for mothers during quiet hours. During these 2 hours, lights were dimmed, and staff were asked to minimize entering the patient’s room. Additional protected quiet hours were available upon request. Education incorporated environmental safety protocols, which included ensuring call light accessibility and minimizing the use of monitoring equipment or intravenous fluids when appropriate to reduce obstacles in the room.

#### Reporting and Debriefing Infant Falls and Near Misses

Falls were reported through the hospital’s safety reporting system, following established policies. We created a protocol for near-miss events, defining near misses as any infant found in the arms of a sleeping caregiver. Near-miss incidents are reported to the Falls Committee and unit leadership for review of the events and reinforcement of best practices. Reporting through daily safety briefings was implemented with department leadership to promote ongoing awareness and adherence to protocols.

#### Postfall Investigation, Treatment, and Support

Although not a formal component of the bundle, we have a standardized approach to address infant falls when they occur. Per organizational protocol, a trauma consultation is requested after each infant fall to provide expert assessment, intervention, and monitoring. A physician specializing in trauma medicine will assess the infant and determine subsequent treatment. Our spiritual care services and customer service provide additional support and follow-up to the family. For our healthcare providers, unit leadership follows up with those directly involved to gather information and provide support. This practice can help reduce emotional distress and increase psychological safety, thereby reinforcing a just culture within the organization.

### Measures

The primary outcome measure was the incidence of newborn falls. Additional measures included tracking near-miss events, whereas process measures focused on compliance with the rounding plan and the use of support pillows. It was not feasible to measure and report all bundle elements. However, ensuring compliance with the rounding plan and support pillow use was important because these were new practices.

Safe sleep champions monitored adherence to rounding protocols and the use of supportive pillows by using a standard data collection tool during their regularly scheduled shifts for patients aged 30 days or younger. Audit results were recorded in a Research Electronic Data Capture database for monthly review, and real-time education was provided to address any noncompliance issues. Data were analyzed, reported, and visualized using QI Macros for Excel.

## RESULTS

**Fig. 4.**
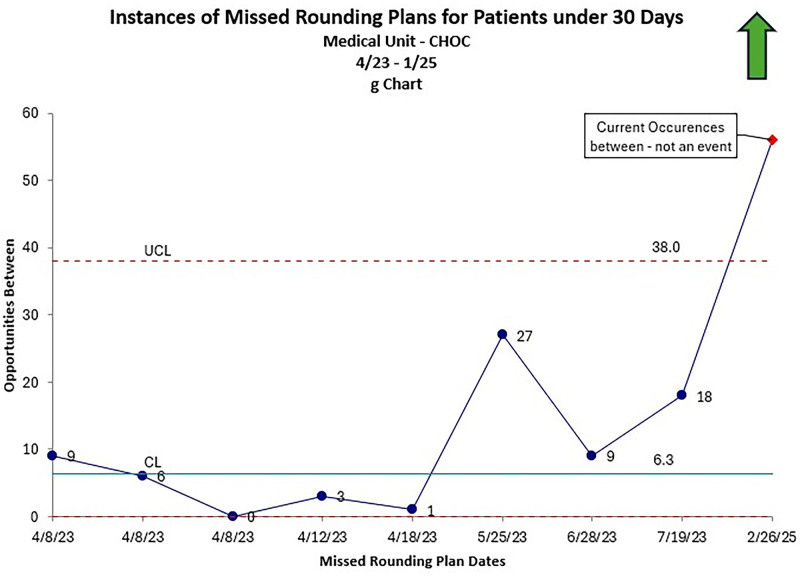
G-chart for rounding plan.

**Fig. 5.**
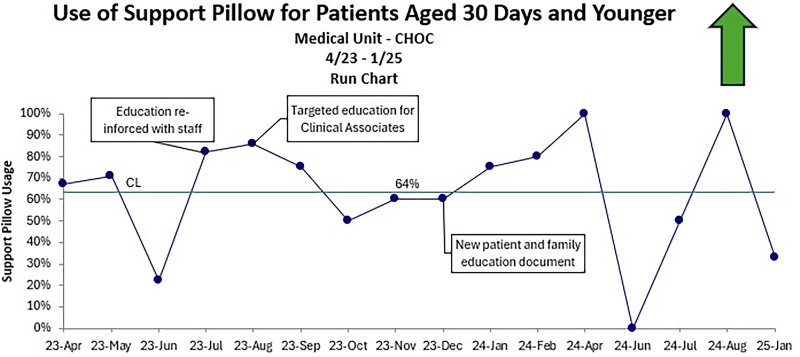
Run chart for support pillow use.

Zero newborn falls and 10 near-miss events have been reported following the implementation of the infant safety bundle from April 2023 to May 2025. Initial compliance rates showed 90% adherence to creating a rounding plan with the family and 67% compliance with supportive pillow use upon implementation (Figs. 4 and 5). However, compliance with the rounding plan dropped to 86% in May and pillow compliance fell to 20% in June. We initiated cycles of improvement, with staff receiving additional education in July. By August 2023, compliance with creating a rounding plan increased to 100% and has been sustained. Supportive pillow compliance improved to 90% in August and fluctuated between 0% and 100% in the months that followed.

## DISCUSSION

Implementing a multifaceted infant fall prevention bundle at our children’s hospital may have helped prevent newborn falls, underscoring the importance of comprehensive intervention strategies. These findings align with evidence supporting the value of bundled approaches in addressing this issue across diverse healthcare settings. To further enhance safety, it is essential to combat the stigma associated with newborn falls by fostering awareness of common risk factors and prioritizing education for healthcare staff and families.

A critical element of our fall prevention program was the engagement of interdisciplinary healthcare teams. Education for staff and consistent reinforcement of safe practices among caregivers promote a culture of vigilance. Previous studies suggest effective family education can be delivered through multiple channels, including verbal discussions, written materials, signage, and safety contracts, ensuring accessibility and clarity for all caregivers.^1,4,7,9,11–13,17,19,20,23,25,21,28^

Other key components of our program included caregiver education and frequent rounding. Most successful programs incorporate staff and parent education^9–15,17,23,25,21^ alongside visual reminders^10,13,14,23,28^ such as posters and crib cards. For example, Carr et al^9^ used a system-wide approach that included safety signage, patient education videos, and increased rounding, achieving a downward trend in newborn falls despite challenges in isolating the most impactful components. Similarly, intentional and frequent rounding has emerged as a pivotal strategy,^9,11,13,14,20,23^ given that maternal and environmental risk factors can be addressed simultaneously. During rounds, staff can address caregiver fatigue, transfer infants to cribs, and ensure the accessibility of call lights, fostering a proactive safety culture. Lipke et al^23^ significantly reduced infant falls and near-miss events by integrating caregiver education, safety agreements, increased rounding, and standardized reporting protocols.

Additionally, we used HDFS, which can provide an additional layer of safety by identifying high-risk dyads.^12,14,17,20,21^ However, challenges persist in predicting sudden caregiver fatigue. For instance, Ainsworth et al^17^ noted a reduction in falls after implementing an assessment tool and safety protocols. Still, they observed a subsequent increase in incidents attributed to caregivers falling asleep unexpectedly. This observation underscores the importance of ongoing evaluation and refinement of assessment tools to improve predictive accuracy. Alternatively, Carr et al^9^ chose not to use a fall risk assessment tool and instead considered every infant at high risk. Because exhausted caregivers can fall asleep suddenly and without warning, the importance of having an alert adult remain in the room during infant holding and feeding cannot be overemphasized.^12,17,18,20,23^

We also established standardized reporting and debriefing processes to identify infant fall trends, contributing factors, and near-miss events. For example, Unal et al^16^ found that near-miss events preceded most newborn falls, emphasizing the importance of tracking these events to inform targeted interventions. Similarly, Loyal et al^15^ observed sustained reductions in fall rates following heightened awareness and improved incident reporting, which provide actionable insights for refining prevention strategies.

Maternal rest is another key component of safety bundles, as caregiver fatigue is a common precipitant of newborn falls. Programs incorporating quiet times, safe sleep contracts, and respite nurseries have shown substantial reductions in fall rates. Karlsson et al^10^ and Galuska^13^ reported 0 falls after implementing maternal rest-focused interventions. Knipper et al^20^ further demonstrated the value of addressing caregiver fatigue combined with positive framing of near-miss events and celebrating program successes.

It should be noted that a cesarean section alone is not a causal risk factor for infant falls and that all infants should be considered at risk, regardless of delivery type. A combination of interrelated factors, such as sleep deprivation, pain medication, breastfeeding, and time of day, contributes to the risk of infant falls.

Implementing innovative strategies, such as using the support pillow during holding and feeding, may require more effort. We found that additional education was needed to adopt the support pillow. During audits, caregivers of nonbreastfed (bottle or gavage-fed) infants seemed less likely to use the pillow. It is possible that staff and caregivers underestimated its importance across different types of feeding methods. The exact reasoning for this finding is unknown and warrants further exploration. Reeducation with staff focused on placing the pillow in the room before admission, revising instructional materials for caregivers with pictures of the pillow in use, and reinforcing pillow use for all feeding methods. With these measures, the support pillow was well received. Miner^11^ observed a significant reduction in falls after introducing a supportive pillow as part of a broader safety bundle, suggesting its potential to enhance caregiver stability and comfort. It remains unknown if a support pillow is crucial for bundle success, highlighting the need for further exploration.

The implementation of the supportive pillow should be handled carefully to prevent inappropriate use. Caregivers should be aware that placing a pillow in the crib poses a risk to infant safety. Staff should monitor the use of pillows to ensure alignment with safe sleep practices.

## STRENGTHS AND LIMITATIONS

Several limitations to this initiative have been identified in the literature. Most of the research on infant fall prevention comprises QI initiatives, which means that causal inferences cannot be drawn, and alternative explanations for intervention effects cannot be ruled out. Most studies are conducted at a single site, so results cannot be generalized to other settings.

One challenge in evaluating bundled interventions is that it remains unknown which components are responsible for producing their effects. However, addressing multiple risk factors through a bundled approach is more likely to have an impact than single interventions. Future work is recommended to investigate whether interventions require higher degrees of effort to implement and ensure compliance, which is key to the success of the bundle, specifically related to the supportive pillow, given that compliance with this component was not consistent.

Although quiet hours were promoted, staff did not enforce that caregivers rest during this time. Parents were able to choose how to use this time, including the use of their electronic devices. Additionally, staff were unable to control the timing of urgent patient needs during quiet hours.

A limitation of this initiative was the reliance on a manual auditing process. Safe sleep champions completed audits during their shifts. Competing patient care priorities made it challenging for staff members to complete the audit consistently. We are currently in the process of transitioning to automated data collection to reduce existing gaps.

Before implementing our new bundle, infant falls and near misses were likely underreported, making it difficult to compare results to baseline values. There can be long periods between events, making it difficult to determine associations among interventions, infant falls, and near misses. Infant fall events are relatively rare, making statistical analysis challenging. In addition, outcomes of fall prevention programs are evaluated using different metrics across organizations (eg, total number of incidents, rate of falls per patient births, occurrence of near-miss events), making it difficult to compare our results with previous reports.

## CONCLUSIONS

Pediatric hospitals are charged with protecting the health and safety of the children in their care, which includes implementing evidence-based strategies to reduce the risk of infant falls. Yet, there is little evidence of strategies specific to the pediatric hospital setting. This initiative demonstrated that implementing a baby drop prevention bundle may have mitigated this risk. It represents a step forward in developing safety precautions specific to newborns at children’s hospitals. A single newborn fall is one too many due to the serious consequences for the patient, family, and staff.

## Supplementary Material


