# Supplementary material for: An Initiative to Prevent Newborn Drops through the Implementation of a Prevention Bundle at a Children’s Hospital
Source: Pediatr Qual Saf. 2026 Jul 29;11(4):e836. doi: 10.1097/pq9.0000000000000836 (PMC13423378; doi:10.1097/pq9.0000000000000836)
Supplement: Supplementary file 1 [file pqs-11-e836-s001.pdf]

IMPORTANT INFORMATION FROM CHOC

Preventing Infant Falls/Drops

An infant drop is when a baby being held or carried, falls or slips from a person's hands, arms, lap , etc. In the United States alone, about 600 to1,600 newborns have an infant fall/drop event while in the hospital.

Some factors that can lead to infant fall/drop events are:

- Caregiver exhaustion, especially in a postpartum mother
- Breastfeeding
- More than 2 days postpartum
- Early morning hours (midnight to 7:00)
- Caregiver (especially postpartum mother) recent use of sedating medications
- Cesarean birth
- Thinking your infant is in a different position than they actually are

It is easy to fall asleep when cuddling or feeding your baby. Please be aware that infants have been dropped in the past when a caregiver has fallen asleep.

How can we partner to prevent Infant Falls/Drops?

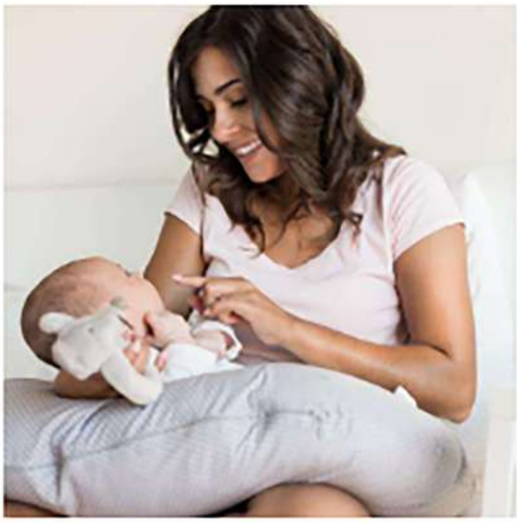

- Evidence shows use of a support pillow/device while breastfeeding, bottle feeding or holding your child can help decrease the risk of an infant fall/drop. You may bring in your home support pillow/device to use while in the hospital.
- Keep the call light close when feeding your child so you can call for help if you need to.
- Place your baby safely on their back in their crib if you feel sleepy while holding or feeding your baby.
- During night feedings have a support person stay with you to help keep you alert.
- Reach out to a CHOC associate or support person to assist you if you are unable or too tired to get up safely and put your baby in the crib.
- Always make sure that anyone holding your baby is awake and alert.
- If you accidentally fall asleep while feeding or holding, a CHOC associate will move your baby to their crib without disturbing your rest.
- If your baby falls or is accidentally dropped, you may pick your baby up then immediately call a CHOC associate by either using the call light or calling from the door to get help.

Reviewed 9/2023
